# Structure–Property Relationships in Zwitterionic Pyridinium–Triazole Ligands: Insights from Crystal Engineering and Hirshfeld Surface Analysis

**DOI:** 10.3390/ijms26115123

**Published:** 2025-05-27

**Authors:** Gerzon E. Delgado, Jonathan Cisterna, Jaime Llanos, Ruth Pulido, Nelson Naveas, Pilar Narea, Pilar Amo-Ochoa, Félix Zamora, Yasna León, Iván Brito

**Affiliations:** 1 Departamento de Química, Facultad de Ciencias, Universidad de los Andes, Mérida 5101, Venezuela; gerzon@ula.ve; 2 Departamento de Química, Facultad de Ciencias, Universidad de Católica del Norte, Sede Casa Central, Av. Angamos 0610, Antofagasta 1270709, Chile; jonathan.cisterna@ucn.cl (J.C.); jllanos@ucn.cl (J.L.); 3 Departamento de Química, Universidad de Antofagasta, Av. Universidad de Antofagasta 02800, Campus Coloso, Antofagasta 1240000, Chile; ruth.pulido@uantof.cl (R.P.); pilar.narea@uantof.cl (P.N.); yasna.leon@uantof.cl (Y.L.); 4 Instituto Universitario de Ciencia de Materiales “Nicolás Cabrera” (INC), Universidad Autónoma de Madrid, Campus de Cantoblanco, 28049 Madrid, Spain; nelson.naveas@estudiante.uam.es; 5 Departamento de Física Aplicada, Universidad Autónoma de Madrid, 28049 Madrid, Spain; 6 Departamento de Ingeniería Química y Procesos de Minerales, Universidad de Antofagasta, Avenida Angamos 601, Antofagasta 1270300, Chile; 7 Departamento de Química Inorgánica, Universidad Autónoma de Madrid, 28049 Madrid, Spain; pilar.amo@uam.es (P.A.-O.); felix.zamora@uam.es (F.Z.); 8 Institute for Advanced Research Chemistry (IAdChem), Universidad Autónoma de Madrid, 28049 Madrid, Spain; 9 Condensed Matter Physics Center (IFIMAC), Universidad Autónoma de Madrid, 28049 Madrid, Spain

**Keywords:** non-covalent interactions, crystal engineering, zwitterionic ligands, hydrogen bond networks, energy framework, Hirshfeld surface analysis, proton conduction, positional isomerism, DFT

## Abstract

This article discloses the synthesis of four new positional isomeric zwitterionic ligands exhibiting semi-flexible and flexible characteristics—*n*-pyridinium-1,2,3-triazole-4-carboxy-5-Acetate (*n*-PTCA), and *n*-methylpyridinium-1,2,3-triazole-4-carboxy-5-Acetate (n-MPTCA; where *n* = 3, 4)—which were derived from an aqueous solution of the corresponding sodium salts in an acidic medium (HCl). These compounds are successfully synthesized and characterized with FT-IR and multinuclear NMR spectroscopy; likewise, proper single crystals are obtained for each compound. All compounds adopt zwitterionic forms in the solid state, which are stabilized via intermolecular proton transfer processes involving HCl and solvent molecules. A single-crystal X-ray analysis revealed how positional isomerism and molecular flexibility influence the supramolecular topology. Specifically, 3-PTCA and 4-PTCA exhibit isomorphic hydrogen bond networks, while 3-MPTCA and 4-MPTCA display distinct packing motifs, attributed to the presence of a methylene spacer between the pyridinium and triazole rings. The Hirshfeld surface analysis quantitatively confirmed the dominance of O···H/H···O and N···H/H···N interactions in the solid-state architecture. These strong hydrogen-bonding networks are indicative of the potential proton-conductive behavior in the crystalline state, positioning these compounds as promising candidates for applications in proton-conducting materials. The structural insights gained underscore the pivotal role of molecular topology in tailoring crystal packing, with implications for the rational design of zwitterionic ligands in functional materials, including MOFs and coordination polymers. The calculated HOMO-LUMO energy gaps reveal a significant electronic variability among the ligands, influenced primarily by the positional isomerism and structural flexibility introduced by the methylene spacer.

## 1. Introduction

N-heterocycles play a key role in biological and synthetic chemistry, forming the backbone of nucleic acid structures and many pharmacologically active molecules [1,2]. Among them, azoles—particularly 1,2,3-triazoles—have garnered significant attention due to their chemical stability, electronic properties, and synthetic versatility [3]. The Cu(I)-catalyzed azide–alkyne cycloaddition (CuAAC), a prototypical “click” reaction, provides efficient and regioselective access to 1,4-disubstituted triazoles, making them valuable scaffolds in drug discovery, catalysis, and materials science [4,5].

Beyond their molecular reactivity, 1,2,3-triazole-based ligands have demonstrated significant potential in the design of multitopic coordination polymers (CPs) and metal–organic frameworks (MOFs), owing to their multidentate coordination modes and tendency to form directional hydrogen bonds [6,7,8]. Recent studies have explored their incorporation into functional materials such as corrosion inhibitors, luminescent complexes, and photovoltaic devices [9,10,11]. Their nitrogen-rich frameworks and geometrical rigidity render them excellent candidates for constructing ordered supramolecular architectures.

Concurrently, zwitterionic ligands—bearing spatially separated positive and negative charges—have emerged as promising building blocks in crystal engineering [12,13,14]. These molecules can form robust hydrogen bond networks and electrostatic interactions, favoring the self-assembly of extended crystal structures [15,16,17]. Despite their potential, the deliberate use of zwitterions in engineering supramolecular networks and coordination materials remains underexplored, particularly regarding the role of positional isomerism and molecular flexibility.

From a crystal engineering perspective, molecular design strategies that modulate parameters such as the spacer length, substitution pattern, and charge distribution allow precise control over intermolecular interactions [18,19,20]. These, in turn, influence crystal packing, hydrogen-bonding topology, and emergent functional properties—including proton conduction [21,22]. The synergy between positional isomerism and supramolecular organization has proven crucial for tailoring material behavior, particularly in hydrogen-bond-mediated proton-conducting solids [23,24,25].

Zwitterionic materials have recently attracted growing interest for their proton-conducting capabilities, making them promising candidates for applications such as proton-exchange membrane fuel cells (PEMFCs) and solid-state electrolytes. For instance, zwitterion-functionalized nanofiber composites have demonstrated an enhanced proton mobility due to continuous hydrogen bond pathways [26,27,28], while zwitterionic polymers have shown competitive proton conductivities and an improved stability under humid conditions [29,30,31]. A recent study reported a novel zwitterion-based membrane applied successfully in PEM fuel cells, highlighting their potential as next-generation functional materials [32,33,34].

Herein, this work reports the synthesis, crystal structures, and supramolecular properties of four novel zwitterionic ligands based on *n*-pyridinium-1,2,3-triazole-4-carboxy-5-Acetate (*n*-PTCA) and *n*-methylpyridinium-1,2,3-triazole-4-carboxy-5-Acetate (*n*-MPTCA; where n = 3,4). These compounds, obtained under acidic aqueous conditions from their corresponding sodium salts, exhibit either semi-flexible or flexible molecular backbones. The presence of a methylene spacer in selected isomers introduces conformational adaptability, which directly impacts the crystal packing and hydrogen bond arrangements.

Through a single-crystal X-ray diffraction and Hirshfeld surface analysis, we examine how isomeric variation and ligand flexibility influence non-covalent interaction patterns and the supramolecular architecture. Special attention is given to the role of hydrogen bond networks as potential proton conduction pathways, emphasizing the promise of these zwitterionic systems for crystal engineering and functional materials applications.

## 2. Results and Discussion

### 2.1. Syntheses

The zwitterionic ligands 3-, 4-PTCA, and 3-, 4-MPTCA were synthesized via a stepwise sequence starting from their corresponding ester precursors, which were obtained through a [3 + 2] dipolar cycloaddition between *n*-pyridyl azides (3-PTCA and 4-PTCA) or *n*-(azidomethyl)pyridine (3-MPTCA and 4-MPTCA) and diethyl 1,3-acetonedicarboxylate, following previously reported procedures [35,36,37,38]. The resulting esters were saponified with aqueous NaOH to generate corresponding sodium salts in quantitative yields. The subsequent acidification with dilute HCl (0.1 N) led to the formation of the zwitterionic ligands described above as hygroscopic solids, which were stored under dry or inert conditions to avoid degradation.

**Scheme 1:**
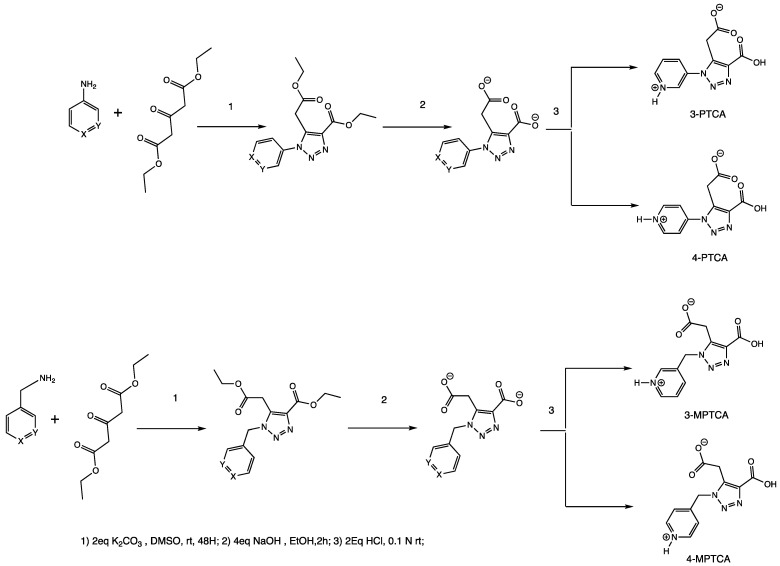
General synthetical route of zwitterionic positional isomers.

The final compounds are highly soluble in water and moderately soluble in protic solvents, such as ethanol and hot methanol. Their general synthetic pathway is depicted in Scheme 1, which highlights the three-step protocol involving the cycloaddition, base hydrolysis, and acid-mediated zwitterion formation.

### 2.2. Crystal Structure

The crystal data, data collection, and refinement parameters for all compounds are shown in Table 1.

The single-crystal X-ray diffraction confirmed that all four compounds crystallize in the zwitterionic form, with the positively charged pyridinium nitrogen and a deprotonated carboxylate group. The formation of these internal ion pairs likely results from the intermolecular proton transfer from both the carboxylic acid moiety and hydrochloric acid to the solvent and nitrogen sites. 3- and 4-PTCA crystallize in the orthorhombic space group *Pna*2_1_, while 3-MPTCA adopts the orthorhombic *P*2_1_2_1_2_1_ and 4-MPTCA the monoclinic *P*2_1_/*c* space group, all with Z = 4. These space groups reflect the influence of both positional isomerism and structural flexibility on the packing arrangement and intermolecular interactions. Bond distances and angles across all structures fall within typical values [39] and are consistent with CSD reference standards [40]. Proton positions were refined using a riding model, and both N–H and O–H groups participate in an extensive hydrogen-bonding network, where O- and N- bound H atoms were initially located in a difference Fourier map and were then added in idealized positions and further refined according to the riding model with O−H = 0.82 Å and N−H =0.86 Å and with U_iso_(H) = 1.5U_eq_(O) or 1.2U_eq_(N).

**Figure 1:**
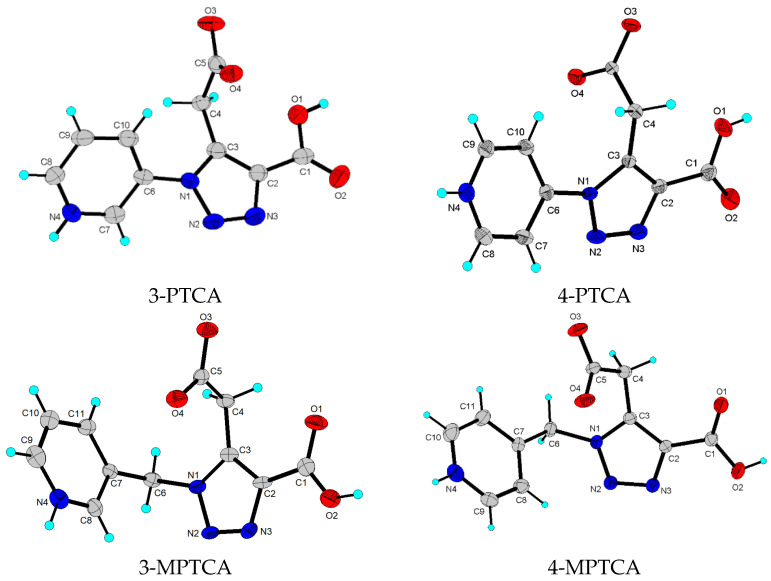
The asymmetric unit for all compounds with an anisotropic ellipsoid representation, together with an atom labeling scheme. The ellipsoids are drawn at a 30% probability level. Hydrogen atoms are depicted as spheres with arbitrary radii.

Figure 1 illustrates the asymmetric units of all compounds, emphasizing the conformational variability introduced by the methylene spacer in 3- and 4-MPTCA. Dihedral angles between the triazole, pyridinium, and carboxylate groups (Table 2) reveal notable differences in the molecular geometry among isomers. These torsional differences serve as a foundation for understanding the observed diversity in the supramolecular assembly.

A CSD search revealed no previously reported zwitterionic structures for this specific triazole–pyridinium–carboxylate framework, highlighting the novelty of the present ligands.

Solid-state studies show the cooperative actions of non-covalent interactions. The molecular structure and crystal packing of all compounds are stabilized by intermolecular O−H···O, N−H···O, and O−H···O/N−H···O (O-atom carboxylate group) hydrogen bond interactions (see Table 3). In all compounds, the supramolecular structure is established as infinite one-dimensional chains and rings exhibiting a complex binary graph-set with varying degrees of the pattern extending along different directions. These supramolecular structures are held together by hydrogen bond interactions (C−H···N and C−H···O), giving rise to three-dimensional networks in all structures.

**Figure 2:**
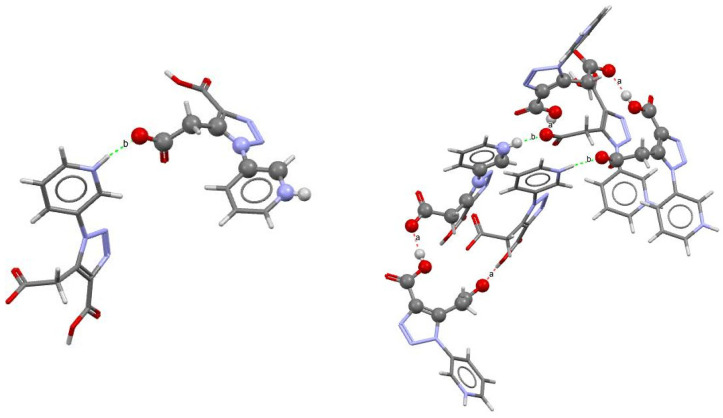
The view shows the chain and ring with the graph-set *motifs* C(9) (b) and $R_{6}^{5}$ (44) (a) as representative examples of the supramolecular structure of 3- and 4-PTCA.

The compounds 3- and 4-PTCA have identical supramolecular structures, known as isographic hydrogen bond patterns [41]. The molecules in these compounds are connected by chains with the graph-set *motifs* of C(8); C(9)$;\;C_{2}^{1}$(11); $C_{2}^{2}$(17); and $C_{4}^{3}$(28) and a non-centrosymmetric ring with a graph-set *motifs* of $R_{6}^{5}$ (44). Figure 2 shows a chain C(9) and a non-centrosymmetric ring $R_{6}^{5}$ (44) as a representative example of the supramolecular structure for these two compounds. The atomic, molecular, supramolecular, and spectroscopic properties of compounds 3- and 4-PTCA are very similar, as well as other physical properties such as solubility and melting points; however, the conformational properties and the fingerprint plots are different, as demonstrated by the dihedral angles indicated in Table 2 and Figures S1–S4, respectively. For further clarity, the isostructurality was quantitatively deciphered from the commonly used method, which uses the unit cell parameters of two crystal structures to calculate the unit cell similarity (Π) [42], when Π=0.02004, as being quite isostructural between them.

**Figure 3:**
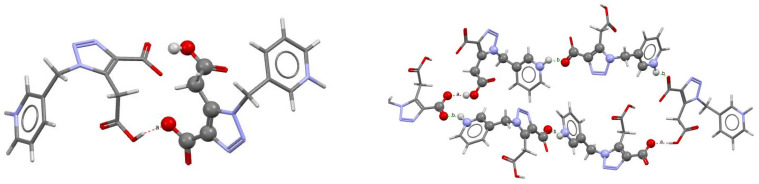
The view shows the chain and ring with the graph-set *motifs* C(8) (b) and $R_{6}^{6}$(52) (a) as a representative example of the supramolecular structure of 3-MPTCA.

For compounds 3- and 4-MPTCA, the supramolecular structures do not exhibit isographic hydrogen bond patterns like those in compounds 3- and 4-PTCA. In the case of 3-MPTCA, the molecules are interconnected by chains and a ring with a graph-set *motifs* of C(8); C(10); $C_{2}^{1}$(14); $C_{2}^{2}$(18); $R_{6}^{6}$(48); and $R_{6}^{6}$(52), respectively. Figure 3 depicts C(8) chains and $R_{6}^{6}$(52) rings, as representative supramolecular structure examples.

**Figure 4:**
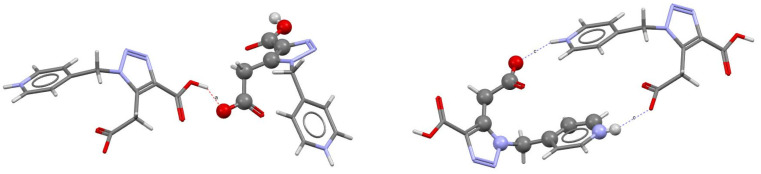
The view shows chains and rings with the graph-set notation C(8) (a) and $R_{2}^{2}$(22) (c) as a representative example of the supramolecular structure of 4-MPTCA.

In the case of 4-MPTCA, the molecules are interconnected through a complex network of hydrogen-bonded chains and rings with the following graph-set *motifs*: C(8); C(11); $R_{2}^{2}$(22); $R_{4}^{4}$(30); $C_{4}^{4}$(34); $R_{4}^{4}$(38); $R_{6}^{6}$(50); $R_{6}^{6}$(56); $C_{2}^{1}$(13); $C_{2}^{2}$(19); $C_{4}^{3}$(32); $R_{6}^{4}$(42); $R_{6}^{5}$(48); $R_{6}^{6}$(54); $C_{1}^{2}$(8); $C_{2}^{2}$(22); $C_{3}^{4}$(30); $R_{4}^{6}$(38); $R_{5}^{6}$(52); and $R_{6}^{6}$(66). Figure 4 shows C(8) chains and $R_{2}^{2}$(22) rings, as a representative supramolecular structure of 4-MPTCA. This compound shows a greater number of supramolecular interactions than its positional isomer, 3-MPTCA. The position of the nitrogen atom on the pyridinium ring in this compound allows for greater supramolecular diversity.

The geometrical parameters corresponding to these non-covalent interactions are summarized in Table 3.

The conformational properties demonstrated by the dihedral angles indicated in Table 2 and the fingerprint plots generated from the crystal structures (Figures S1–S4) account for their non-isostructural character.

### 2.3. Hirshfeld Surface and Topological Studies

**Figure 5:**
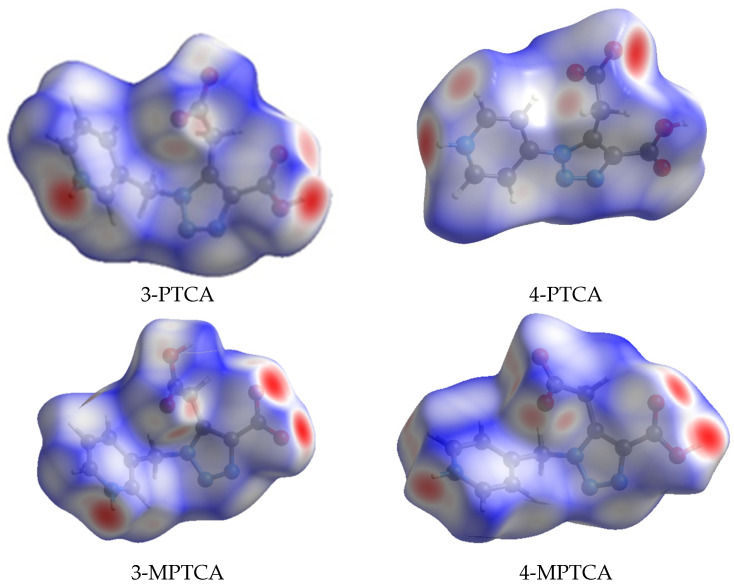
A view of the three-dimensional Hirshfeld surface of all compounds plotted over *d_norm_*. Intermolecular contacts longer than the sum of the van der Waals radii are colored blue, and contacts shorter than the sum of the radii are colored red. Contacts equal to the sum of the radii are white.

Intermolecular interactions in all compounds were verified using the CrystalExplorer software (version 17.5; University of Western Australia: Perth, Australia) [43] through a Hirshfeld surface analysis [44] and two-dimensional fingerprint plots [45]. The normalized contact distance (*d_norm_*) surfaces were generated by mapping the internal (*d_i_*) and external (*d_e_*) distances between surface points and the nearest atoms in the crystal lattice. On these surfaces, short contacts (stronger than the sum of van der Waals radii) are represented in red, contacts close to the sum appear in white, and longer contacts are shown in blue (Figure 5). These visualizations highlight key regions of non-covalent interactions.

The most significant interactions observed across all compounds are O–H···O, N–H···O, and C–H···O hydrogen bonds, which appear as intense red regions on the Hirshfeld surfaces. These contacts correspond to strong directional interactions that govern the supramolecular organization of the crystals and are consistent with the hydrogen bonds observed in the X-ray diffraction analysis.

To gain quantitative insight, fingerprint plots were constructed by correlating the *d_i_* and *d_e_* values in increments of 0.01 Å. These plots allow the decomposition of the surface into contributions from specific contact types (Figures S1–S4). In all structures, O···H/H···O interactions are the dominant contributors to the total surface area, accounting for 39.5–43.7%, followed by N···H/H···N, H···H and C···H/H···C interactions to a lesser extent.

For example, in compound 3-PTCA, the fingerprint plot shows the following contributions: O···H/H···O (42.5%), N···H/H···N (20.1%), H···H (14.1%), C···H/H···C (8.3%), and C···O/O···C (7.8%). Minor contributions include C···C (1.6%), C···N/N···C (2.6%), O···N (2.2%), N···N (0.7%), and O···O (0.1%), which have a minimal directional influence on the molecular packing (Tables S1–S4).

The O···H/H···O interactions are represented in the fingerprint plots as two sharp symmetrical spikes centered around *d_e_* + *d_i_* ≈ 1.5–1.7 Å, indicating strong hydrogen bonding. Similarly, N···H/H···N contacts manifest as symmetrical wings between 2.4 and 2.7 Å, while C···H/H···C contacts—contributing 8.3–11.1%—show broader wings at 3.0–3.3 Å, which is suggestive of possible C–H···π interactions within the crystal packing.

This combined structural and surface analysis confirms the central role of hydrogen bonding and positional isomerism in dictating the supramolecular organization of these zwitterionic ligands.

Finally, the energy framework [46] was analyzed to gain a better understanding of the crystal packing, topology, and supramolecular arrangement. This method allows for the calculation and comparison of different energy components, including repulsion (E_rep), electrostatic (E_ele), dispersion (E_dis), polarization (E_pol), and total energy (E_tot), based on the anisotropy of pairwise intermolecular interaction energies (see Figures S5–S8 and Tables S1–S4). The cylinder thickness represents the strength of the interactions, directly correlating with the energy magnitude and providing insights into the stabilization of the crystal packing [46].

The orientation of the tubes suggests that the framework formation is driven by translational or centrosymmetric elements. However, this arrangement also facilitates the emergence of additional weak interactions within the crystal structure.

**Figure 6:**
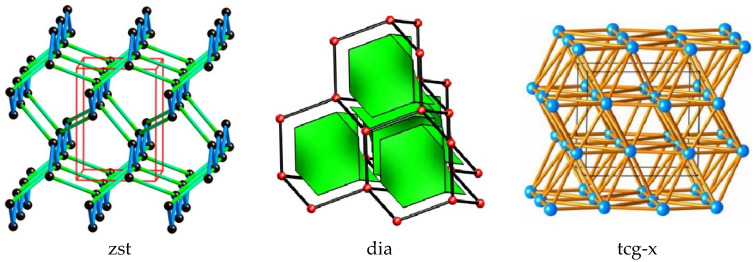
The resulting topologies in all compounds.

The calculations revealed that dispersion interactions form a distorted *zig-zag* ladder-like topology in the compounds with a *zst* topology for compounds 3-PTCA and 4-PTCA, meanwhile compounds 3-MPTCA and 4-MPTCA have a *dia* and *tcg-x* topology, respectively (see Figure 6). A significant difference between E_ele and E_pol was observed, likely due to the presence of classical 1D hydrogen bond interactions.

### 2.4. Density Functional Theory (DFT) Calculations

The electronic structure and reactivity parameters of the synthesized ligands were investigated through DFT calculations. All calculations were performed using the hybrid B3LYP functional in combination with the def2-TZVP basis set (see Section 3.4). This level of theory was selected for its well-established accuracy in describing the electronic properties of organic molecules, particularly frontier molecular orbitals and global reactivity descriptors. The calculated electronic parameters, including frontier molecular orbitals (Highest Occupied Molecular Orbital—HOMO and Lowest Unoccupied Molecular Orbital—LUMO), ionization potentials, electron affinities, electronegativities, hardness, softness, and electrophilicity indices, are presented in Table 4. The calculated HOMO-LUMO energy gaps (E_gap_) for ligands were found to be 1.88 eV (3-PTCA), 2.02 eV (4-PTCA), 4.04 eV (3-MPTCA), and 3.10 eV (4-MPTCA). These values reveal significant electronic variability among the ligands, influenced primarily by the positional isomerism and structural flexibility introduced by the methylene spacer. Ligand 3-MPTCA exhibited the largest HOMO-LUMO gap, indicating a higher kinetic stability and lower chemical reactivity compared to ligands 3-PTCA, 4-PTCA, and 4-MPTCA. Conversely, ligand 3-PTCA displayed the narrowest HOMO-LUMO gap, suggesting a greater chemical reactivity and potential ease in forming coordination bonds. The global hardness (η), reflecting the resistance to electron transfer, varied accordingly, with 3-MPTCA having the highest hardness value (2.02 eV), which is consistent with its large HOMO-LUMO gap. On the other hand, ligand 3-PTCA exhibited the lowest hardness value (0.94 eV), corroborating its higher chemical reactivity. Additionally, ligand 3-MPTCA had the lowest electrophilicity index (4.53 eV), indicating the lowest propensity to accept electrons, while ligand 3-PTCA had the highest electrophilicity index (11.18 eV), marking it as the most electrophilic of the set.

**Figure 7:**
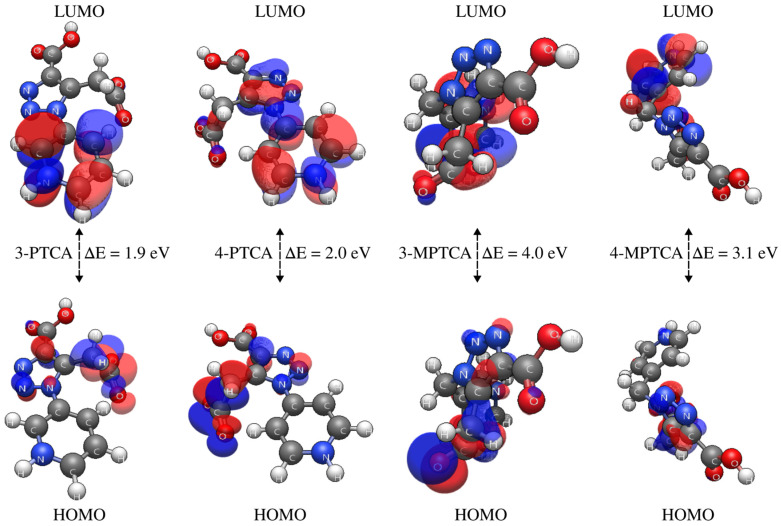
Visualizations of the HOMO and LUMO molecular orbitals for the synthesized ligands. In the isosurfaces, the red region indicates a high electron concentration, whereas the blue region represents a low electron concentration.

Figure 7 displays the computed frontier molecular orbitals (HOMO and LUMO) obtained by DFT calculations for all ligands. In general, the HOMO is primarily localized on the triazole nitrogen atoms and the carboxylate oxygens, regions associated with electron-rich sites capable of coordination. Conversely, the LUMO predominantly encompasses the pyridinium and triazole rings, reflecting an electron-accepting character. The presence of the flexible methylene spacer in 3-MPTCA and 4-MPTCA slightly disrupts the extended conjugation, inducing subtle but meaningful differences in the orbital distribution compared to their more rigid analogs (3-PTCA and 4-PTCA). These differences suggest that both positional isomerism and ligand flexibility significantly modulate the electronic structure and coordination potential of these promising N/O mixed ligands.

## 3. Materials and Methods

### 3.1. Chemicals

Chemicals were A.R. grade and used without further purification (hydrochloric acid: 32%, chemically pure grade, Sigma-Aldrich, St. Louis, MO, USA). The syntheses of the ester precursor and the sodium salt of the carboxylate ligands were carried out according to the procedure previously described and reported [38].

### 3.2. Characterization

(3-PTCA) Colorless parallelepipeds, m.p. 241–242 °C, FT-IR (KBr pellet, cm^−1^): 3482(w) ν(N–H), 3401(w) (O–H), (3070(w), 3048(w) ν(Csp^2^–H) 2939(vw), 2925(vw) ν(Csp^3^–H),1467(m), ν(C=O and/or C=C), 1618(s), ν(C–N)1410(m), ν(N=N and/or -CH_3_), 1260(vw), ν(C–O), 1260(w), ν(Csp^2^–N), 551 (vw); ^1^H-NMR (300 MHz, DMSO-d^6^) δ 8.82 (dd, J = 4.8, 1.5 Hz, 1H, H2), 8.79 (d, J = 2.5 Hz, 1H, H4), 8.07 (ddd, J = 8.2, 2.6, 1.5 Hz, 1H, H5), 7.71 (dd, J = 8.2, 4.8 Hz, 1H, H6), 4.09 (s, 1H, -CH_2_-);^13^C-NMR (75 MHz, DMSO-d^6^) δ 169.31 (C11), 162.23(C9), 151.45(C4), 146.03(C2), 137.92 (C9-ipso), 137.29 (C1-ipso), 133.51(C6), 132.19(C8-ipso), 124.77(C5), and 29.69(-CH_3_ Acetic acid). (See Figures S9 and S10).

(4-PTCA) Colorless parallelepipeds, m.p. 244–245 °C, FT-IR (KBr pellet,cm^−1^): 3482(w) ν(N–H), 3195(vs) ν(O–H), 3195(w), 3057(m) ν(Csp^2^–H), 2990(w), 2927(m) ν(Csp^3^–H), 1614(vs) ν(C=O and/or C=C), 1597(s) ν(C–N), 1426(m) ν(N=N and/or -CH_3_), 1290(m) ν(C–O), 1248(w) ν(Csp^2^–N), 558 (vw); ^1^H-NMR (300 MHz, DMSO-d^6^) δ 2.51 (d, J = 6.1 Hz, 7H), 4.20 (s, 1H), 7.70 (d, J = 5.2 Hz, 1H), 8.88 (d, J = 5.0 Hz, 1H); ^13^C-NMR (75 MHz, DMSO-d^6^) δ 30.16 (C10), 119.60 (C14, C18), 137.15 (C1, C2), 142.68 (C6), 152.12 (C15, C17), 162.48 (C7), 169.58 (C11). (see Figures S11 and S12).

(3-MPTCA) Colorless flat needle, m.p. 234–235 °C, FT-IR (KBr pellet, cm^−1^): 3482(w) ν(N–H), 3459(vs) ν(O–H), 3112(w), 2993(w) ν(Csp^2^–H), 2930(m), 2993(w), ν(Csp^3^–H),1603(w),1637 (m) ν(C=O and/or C=C), 1637(m), ν(C–N), 1482(m) ν(N=N and/or -CH_3_), 1367(w) ν(C–O), 1221(s), ν(Csp^2^–N), 501 (m); ^1^H -NMR (300 MHz, DMSO) δ 4.21 (s, 2H, 16), 5.76 (s, 2H, 6), 7.44 (ddd, J = 7.9, 4.8, 0.8 Hz, 1H, 11), 7.73 (dt, J = 7.9, 2.0 Hz, 1H, 12), 8.54–8.65 (m, 2H, 13, 15); ^13^C-NMR (75 MHz, DMSO) δ 169.66 (C17), 162.66 (C7), 149.70 (C15), 149.54 (C13), 137.93 (C11), 136.86 (C1), 136.34 (C2), 131.45 (C10), 124.18 (C12), 48.90 (C6), and 29.55 (C16). (See Figures S13 and S14).

(4-MPTCA) Colorless flat needle, m.p. 239–240 °C, FT-IR (KBr pellet, cm^−1^): 3482(w) ν(N–H), 3461(vs) ν(O–H), 3193(w), 3019(w) ν(Csp^2^–H), 2936(w), 2990(m) ν(Csp^3^–H),1614(vs), 1597(s) ν(C=O and/or C=C), 1426(vs) ν(C–N), 1278(m) ν(N=N and/or -CH_3_), ν(C–O), ν(Csp^2^–N), 554(vw); ^1^H-NMR (300 MHz, DMSO-d^6^) δ 3.87 (s, 8H, 16), 4.15 (s, 0H), 5.80 (s, 0H, 6), 7.20–7.28 (m, 0H, 12, 14), 8.55–8.64 (m, OH, 11, 15); ^13^C -NMR (75 MHz, DMSO-d^6^) δ 29.55 (C16), 50.06 (C6), 122.79 (C11, C15), 137.18 (C2), 138.05 (C1), 144.63 (C10), 150.27 (C12, C14), 162.65 (C7), and 169.58 (C17). (See Figures S15 and S16).

FT-IR spectra in the range 400–4000 cm^−1^ were recorded on a Thermo Nicolet Avatar 300 spectrometer using KBr pellets (Thermo Scientific, Waltman, MA, USA). Colorless crystals of the title compounds suitable for X-ray diffraction analysis were selected and measured. Diffraction data were collected at 295(2) K on a Bruker D8 Venture diffractometer equipped with a Photon-III C14 detector, using graphite monochromated MoKα (λ = 0.71073 Å) and Cu-Kα (λ = 1.54178 Å) radiation (Bruker Co.; Billerica, MA, USA). The diffraction frames were integrated using the APEX4 package [47] and were corrected for absorptions with SADABS [48]. The crystal structures of the title compounds were solved by intrinsic phasing [49] using the OLEX2 software (version 15.0, O.dolomanov; Department of Chemistry, Durham University) [50] and refined with full-matrix least-squares methods based on F2 (SHELXL) [49]. All the non-hydrogen atoms were refined with anisotropic displacement parameters. The hydrogen atoms were included from calculated positions and refined riding on their respective carbon atoms with isotropic displacement parameters. The structures of 3-, 4-PTCA, and 3-MPTCA were solved as merohedral twin specimens with the twin law in the reciprocal space expressed by the matrix [−1 0 0; 0–1 0; 0 0 −1]. All geometrical calculations were performed using the program Platon [51].

### 3.3. Hirshfeld Surface and Topological Analysis

The Hirshfeld surface and corresponding 2D fingerprint plots [45] were computed using CrystalExplorer 17.3 [52], with the crystallographic information file (CIF) as input [44]. The normalized contact distance (*d_norm_*) was determined based on *d_e_*, *d_i_*, and the van der Waals radii (VdW) of atoms, following Equation (1). Here, de and di represent the distances from the Hirshfeld isosurface to the nearest external and internal nuclei, respectively, while VdW values were obtained from the literature [53,54].

(1) $$d_{norm}=\frac{d_{i}-r_{i}^{vdW}}{r_{i}^{vdW}}+\frac{d_{e}-r_{e}^{vdW}}{r_{e}^{vdW}}$$

Additionally, energy framework calculations were performed at the B3LYP/6-31G(d,p) level of theory over a range of ±0.002 a.u. [55], using the TONTO computational package (D. Jayatilaka & D. J. Grimwood; Chemistry, School of Biomedical and Chemical Sciences, University of Western Australia, Crawley, Australia) [56], integrated into CrystalExplorer [55]. Hydrogen bond lengths were normalized to standard neutron diffraction values (C–H = 1.083 Å, N–H = 1.009 Å, and O–H = 0.983 Å). Intermolecular interaction energies for molecular pairs in the crystal packing were calculated at the B3LYP/6-31G(d,p) level within a 3.8 Å radius cluster around the molecule [57,58].

Finally, topological analysis was supported by the topcryst.com [59]. The RCSR three-letter codes [60] were used to designate the network topologies. Those nets, that are absent in the RCSR, are designated with the TOPOS NDn nomenclature [61], where N is a sequence of coordination numbers of all non-equivalent nodes of the net, D is periodicity of the net (D = M, C, L, and T for 0-,1-,2-,3-periodic nets), and n is the ordinal number of the net in the set of all non-isomorphic nets with the given ND sequence. To calculate the underlying nets, we used algorithms3, the application of which for specific structures is discussed in the article [62]. The TTD collection [62] was used to determine the topological type of the crystal structure.

### 3.4. Density Functional Theory Calculations

All DFT calculations were carried out using the ORCA software package (version 6.0.1), developed by Prof. Frank Neese and the Molecular Theory and Spectroscopy Group at the Max-Planck-Institut für Kohlenforschung, Mülheim an der Ruhr, Germany, and distributed by FACCTs GmbH (https://www.faccts.de/orca/, accessed on 21 May 2025) [63,64]. Geometry optimization and vibrational frequency analyses were performed employing the hybrid B3LYP functional with the def2-TZVP basis set. The optimized structures were confirmed as true minima on the potential energy surface by ensuring no imaginary frequencies were observed. Frontier molecular orbitals HOMO and LUMO were computed at the same theoretical level (B3LYP/def2-TZVP). These energies were utilized to calculate the global reactivity parameters (GRPs): ionization potential (IP), electron affinity (EA), global hardness (η), global electronegativity (X), chemical potential (μ), global electrophilicity (ω), and global softness (σ).

## 4. Conclusions

In this study, four novel zwitterionic ligands—3-PTCA, 4-PTCA, 3-MPTCA, and 4-MPTCA—were synthesized and structurally characterized through a single-crystal X-ray diffraction and Hirshfeld surface analysis. All compounds crystallize in the zwitterionic form in the solid state, which is stabilized by strong O–H···O and N–H···O hydrogen bonds that define robust supramolecular networks.

Despite their structural similarities, positional isomerism and the presence of the methylene spacer in the MPTCA ligands significantly influence the crystal packing. While 3-PTCA and 4-PTCA exhibit isostructural hydrogen bond networks, 3-MPTCA and 4-MPTCA display distinct supramolecular topologies, highlighting the impact of molecular flexibility on non-covalent interactions. The dihedral angle variations and graph-set analysis further support these structural differences.

Hirshfeld surface and fingerprint plot analyses revealed that O···H/H···O interactions are the dominant contributors to the crystal cohesion, followed by N···H/H···N and C···H/H···C contacts. These findings underscore the importance of hydrogen-bonding interactions in directing the self-assembly of these zwitterionic ligands.

From a crystal engineering perspective, these results demonstrate how fine-tuning molecular topology through an isomeric variation and spacer incorporation allows for the control over the supramolecular architecture. The presence of well-organized hydrogen bond networks also suggests the potential of these ligands for proton-conducting applications, particularly in the design of zwitterionic metal–organic frameworks (ZW-MOFs) or coordination polymers (CPs). Future work will focus on evaluating the physicochemical properties of these materials, including their proton conductivity under humid conditions.

The calculated HOMO-LUMO energy gaps (E_Gap_) for ligands 3-PTCA, 3-PTCA, 3M-PTCA, and 4-MPTCA were found to be 1.88 eV (3-PTCA), 2.02 eV (3-PTCA), 4.04 eV (3M-PTCA), and 3.10 eV (4M-PTCA). These values reveal a significant electronic variability among the ligands, influenced primarily by the positional isomerism and structural flexibility introduced by the methylene spacer. Ligand 3-MPTCA exhibited the largest HOMO-LUMO gap, indicating a higher kinetic stability and lower chemical reactivity compared to ligands 3-PTCA, 4-PTCA, and 4M-PTCA. Conversely, ligand 3-PTCA displayed the narrowest HOMO-LUMO gap, suggesting a greater chemical reactivity and potential ease in forming coordination bonds.

## Figures and Tables

**Table 1:** Crystal data for all compounds.

	**3-PTCA**	**4-PTCA**
CCDC number	2317647	2317648
Empirical formula	C_10_H_8_N_4_O_4_	C_10_H_8_N_4_O_4_
Formula weight	248.20	248.20
Temperature/K	297	297
Crystal system	orthorhombic	orthorhombic
Space group	*Pna2*_1_ (No 33)	*Pna2*_1_ (No 33)
a/Å	12.0311(14)	13.962(5)
b/Å	17.673(2)	17.017(6)
c/Å	5.0309(4)	4.4663(17)
Volume/Å^3^	1069.7(2)	1061.2(7)
Z’	1	1
Z	4	4
ρ_calc_g/cm^3^	1.541	1.554
μ/mm^−1^	1.052	0.124
F (000)	512	512
Crystal size/mm^3^	0.11 × 0.21 × 0.30	0.15 × 0.20 × 0.38
Radiation	CuKα (λ = 1.54178)	MoKα (λ = 0.71073)
2Θ range for data collection/°	4.4 to 59.2	2.8 to 26.5
Index ranges	−10 ≤ h ≤ 13, −17 ≤ k ≤ 19, −5 ≤ l ≤ 5	−17 ≤ h ≤ 17, −20 ≤ k ≤ 21, −5 ≤ l ≤ 5
Reflections collected	11,775	7489
Independent reflections	1462 [R_int_ = 0.177]	1916 [R_int_ = 0.047]
Data/restraints/parameters	1462/0/166	1916/0/165
Goodness-of-fit on F^2^	1.00	1.30
Flack parameter (x)	0.6(1)	0(3)
Final R indexes [all data]	R_1_ = 0.0693, wR_2_ = 0.1904	R_1_ = 0.0432, wR_2_ = 0.1719
Largest diff. peak/hole/e Å^−3^	0.25/-0.25	0.68/−0.65
	**3-MPTCA**	**4-MPTCA**
CCDC number	2317645	2317646
Empirical formula	C_11_H_10_N_4_O_4_	C_11_H_10_N_4_O_4_
Formula weight	262.23	262.23
Temperature/K	297	297
Crystal system	orthorhombic	monoclinic
Space group	*P*2_1_2_1_2_1_ (No 19)	*P*2_1_/*c* (No 14)
a/Å	4.748(12)	4.9510(7)
b/Å	12.88(4)	19.019(3)
c/Å	18.02(5)	11.9930(16)
β/°	-	93.526(7)
Volume/Å^3^	1102(5)	1127.2(3)
Z’	1	1
Z	4	4
ρ_calc_g/cm^3^	1.581	1.545
μ/mm^−1^	0.124	1.030
F(000)	544	544
Crystal size/mm^3^	0.18 × 0.08 × 0.07	0.19 × 0.06 × 0.05
Radiation	MoKα (λ = 0.71073)	MoKα (λ = 0.71073)
2Θ range for data collection/°	2.8 to 25.0	4.4 to 6.4
Index ranges	−5 ≤ h ≤ 3, −15 ≤ k ≤ 15, −21 ≤ l ≤ 21	−5 ≤ h ≤ 5, −22 ≤ k ≤ 19, −14 ≤ l ≤ 14
Reflections collected	6439	13612
Independent reflections	1902 [R_int_ = 0.211]	1921 [R_int_ = 0.315]
Data/restraints/parameters	1902/0/174	1921/0/173
Goodness-of-fit on F^2^	0.97	1.07
Final R indexes [all data]	R_1_ = 0.0712, wR_2_ = 0.1830	R_1_ = 0.0554, wR_2_ = 0.1139
Largest diff. peak/hole/e Å^−3^	0.24/−0.29	0.33/−0.25

**Table 2:** Dihedral angles for all compounds (°).

	**3-PTCA**	**4-PTCA**
C3-N1-C6-C10	−44 (2)	26.7 (8)
C3-C4-C5-O4	−1.4 (1)	26.7 (6)
C3-C2-C1-O1	−7 (2)	−2.0 (8)
	**3-MPTCA**	**4-MPTCA**
N1-C6-C7-C8	−66 (1)	−39.8 (3)
C3-C2-C1-O1	−4.0 (2)	4.6 (3)
C3-C4-C5-O4	−17 (1)	−19.4 (3)

**Table 3:** Hydrogen bond interactions for all compounds.

Sample	D−H∙∙∙A *	Symmetry Code	D∙∙∙A (Å)	D−H∙∙∙A (°)
**3-PTCA**	O1−H1∙∙∙O3	[1 − x, 1 − y, −1/2 + z]	2.519 (12)	173
	N4−H4∙∙∙O3	[−1/2 + x, 1/2 − y, 1 + z]	2.565 (13)	170
	H1∙∙ O3-C5	[1 − x,1 − y, 1/2 + z]	2.519 (12)	173
	H4∙∙∙O3−C5	[1/2 + x, 1/2 − y, −1 + z]	2.565 (13)	170
**4-PTCA**	O1−H1∙∙∙O3	[−x, −y, 1/2 + z]	2.551 (5)	162
	N4−H4∙∙∙O3	[1/2 + x, −1/2 − y,1 + z]	2.640 (6)	179
	H1∙∙∙O3−C5	[−x, −y, 1/2 + z]	2.551 (5)	163
	H4∙∙∙O3−C5	[−1/2 + x, −1/2 − y, −1 + z]	2.640 (6)	179
**3-MPTCA**	O3−H3∙∙∙O1	[−1/2 + x, 1/2 − y, 1 − z]	2.434 (9)	165
	N4−H4∙∙∙O2	[3/2 − x, 1 − y, −1/2 + z]	2.628 (15)	145
**4-MPTCA**	O2−H2∙∙∙O3	[1 + x, 1/2 − y, ½ − z]	2.450 (3)	133
	N4−H4∙∙∙O1	[1 − x, 1/2 + y, ½ − z]	2.757 (3)	142
	(N)H∙∙∙O1−C1	[1 − x, −1/2 + y,1/2 + z]	2.757 (3)	142
	H2∙∙∙O3−C5	[−1 + x, 1/2 − y, −1/2 + z]	2.449 (3)	133

* D = Donor and A = Acceptor.

**Table 4:** Visualizations of the HOMO and LUMO molecular orbitals for the synthesized ligands. All values are reported in eV.

Parameter	3-PTCA	4-PTCA	3-MPTCA	4-MPTCA
E_HOMO_	−5.5285	−5.6125	−6.3038	−5.7310
E_LUMO_	−3.6468	−3.5904	−2.2618	−2.6355
E_Gap_	1.8817	2.0221	4.0420	3.0955
IP	5.5285	5.6125	6.3038	5.7310
EA	3.6468	3.5904	2.2618	2.6355
Hardness (η)	0.9409	1.0111	2.0210	1.5477
Electronegativity (X)	4.5877	4.6014	4.2828	4.1833
Chemical Potential (μ)	−4.5877	−4.6014	−4.2828	−4.1833
Electrophilicity (ω)	11.1849	10.4710	4.5379	5.6532
Softness (σ)	0.5314	0.4945	0.2474	0.3230

## Data Availability

Data are contained within the article and Supplementary Materials.

## Supplementary Materials

The following supporting information can be downloaded at: https://www.mdpi.com/article/10.3390/ijms26115123/s1. CCDC 2317645-2317648 contains the supplementary crystallographic data of compounds 3-PTCA, 4-PTCA, 3-MPTCA, and 4-MPTCA, respectively. These data can be obtained free of charge from The Cambridge Crystallographic Data Centre via www.ccdc.cam.ac.uk/data_request/cif, accessed on 21 May 2025.
