# Supplementary material for: Structure–Property Relationships in Zwitterionic Pyridinium–Triazole Ligands: Insights from Crystal Engineering and Hirshfeld Surface Analysis
Source: Int J Mol Sci. 2025 May 27;26(11):5123. doi: 10.3390/ijms26115123 (PMC12154730; doi:10.3390/ijms26115123)
Supplement: Supplementary file 1 [file ijms-26-05123-s001.zip › ijms-3607205-supplementary.pdf]

Supplementary Information

Structure–Property Relationships in Zwitterionic Pyridinium–Triazole Ligands: Insights from Crystal Engineering and Hirshfeld Surface Analysis

Gerzon E. Delgado[1], Jonathan Cisterna[2], Jaime Llanos[2], Ruth Pulido[3,4], Nelson Naveas[4,5,6], Pilar Narea[3], Pilar Amo-Ochoa[7,8], Félix Zamora[7,8,9], Yasna León[3] and Iván Brito*[3]

Table of content

**Figure S1.** full two-dimensional fingerprint plots for 3-PTCA showing all interactions.	2
**Figure S2.** full two-dimensional fingerprint plots for 4-PTCA showing all interactions.	2
**Figure S3.** full two-dimensional fingerprint plots for 3-MPTCA showing all interactions.	3
**Figure S4.** full two-dimensional fingerprint plots for 4-MPTCA showing all interactions.	3
**Figure S5.** Energy framework of 3-PTCA	4
**Figure S6.** Energy framework of 4-PTCA	5
**Figure S7.** Energy framework of 3-MPTCA	6
**Figure S8.** Energy framework of 4-MPTCA	7
**Figure S9.** FT-IR spectra of 3-PTCA (black) and ligand (red)	8
**Figure S10.** ^{1}H-NMR and ^{13}C-NMR spectra of 3-PTCA	9
**Figure S11.** FT-IR spectra of 4-PTCA (black) and ligand (red)	10
**Figure S12**^{1}H-NMR and ^{13}C-NMR spectra of 4-PTCA	11
**Figure S13.** FT-IR spectra of 3-MPTCA (black) and ligand (red)	12
**Figure S14.** ^{1}H-NMR and ^{13}C-NMR spectra of 3-MPTCA	13
**Figure S15.** FT-IR spectra of 4-MPTCA (black) and ligand (red)	14
**Figure S16.** ^{1}H-NMR and ^{13}C-NMR spectra of 4-MPTCA	15
**Table S1.** Total energy force diagrams and the details of interaction with symmetry operation (Symop) and color-coded interaction distances between molecular centroids (R) in Å for 3-PTCA	16
**Table S2.** Total energy force diagrams and the details of interaction with symmetry operation (Symop) and color-coded interaction distances between molecular centroids (R) in Å for 4-PTCA	17
**Table S3.** Total energy force diagrams and the details of interaction with symmetry operation (Symop) and color-coded interaction distances between molecular centroids (R) in Å for 3-MPTCA	18
**Table S4.** Total energy force diagrams and the details of interaction with symmetry operation (Symop) and color-coded interaction distances between molecular centroids (R) in Å for 4-MPTCA	19

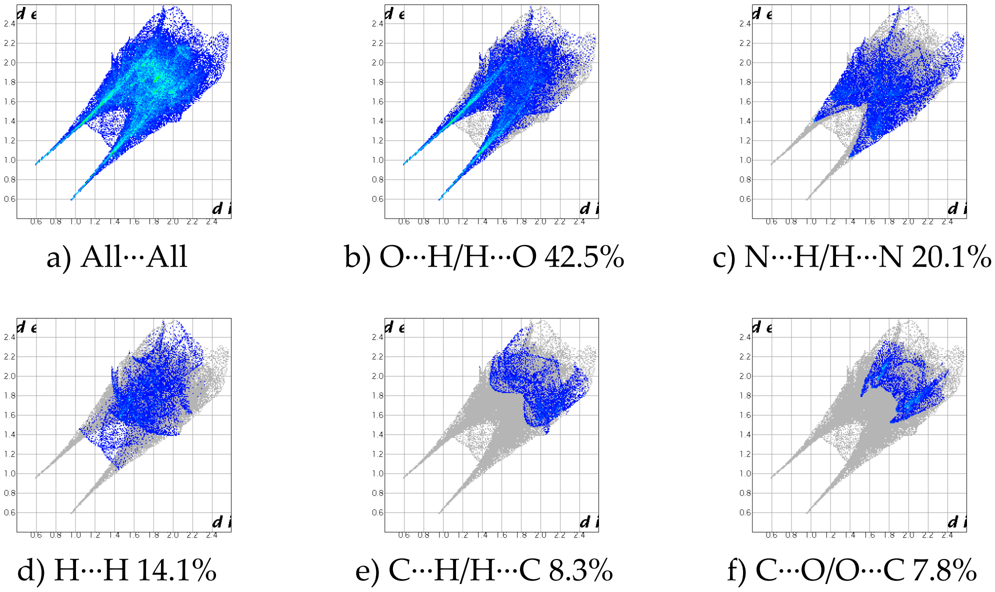

Figure S1. full two-dimensional fingerprint plots for 3-PTCA showing all interactions.

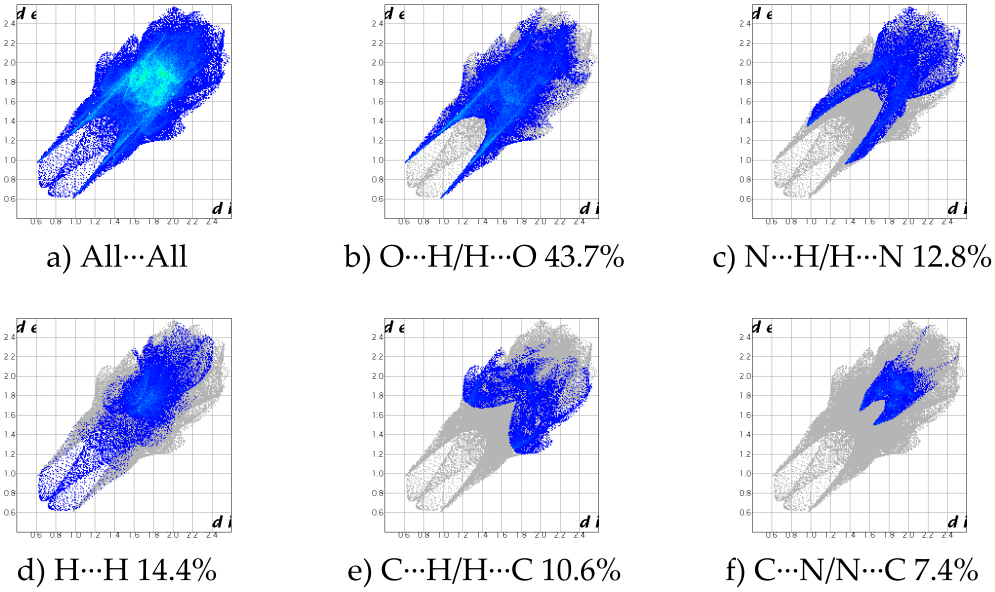

Figure S2. full two-dimensional fingerprint plots for 4-PTCA showing all interactions.

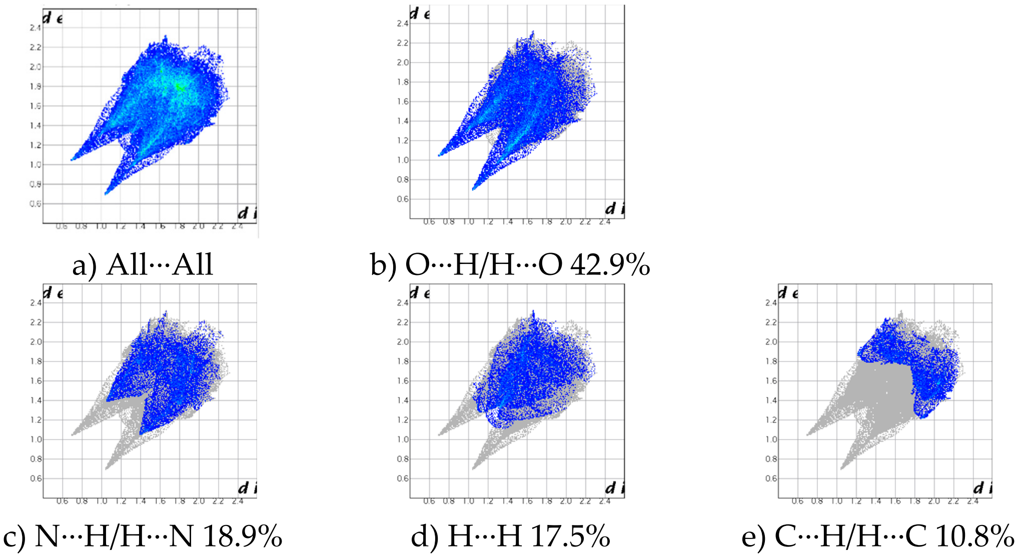

Figure S3. full two-dimensional fingerprint plots for 3-MPTCA showing all interactions.

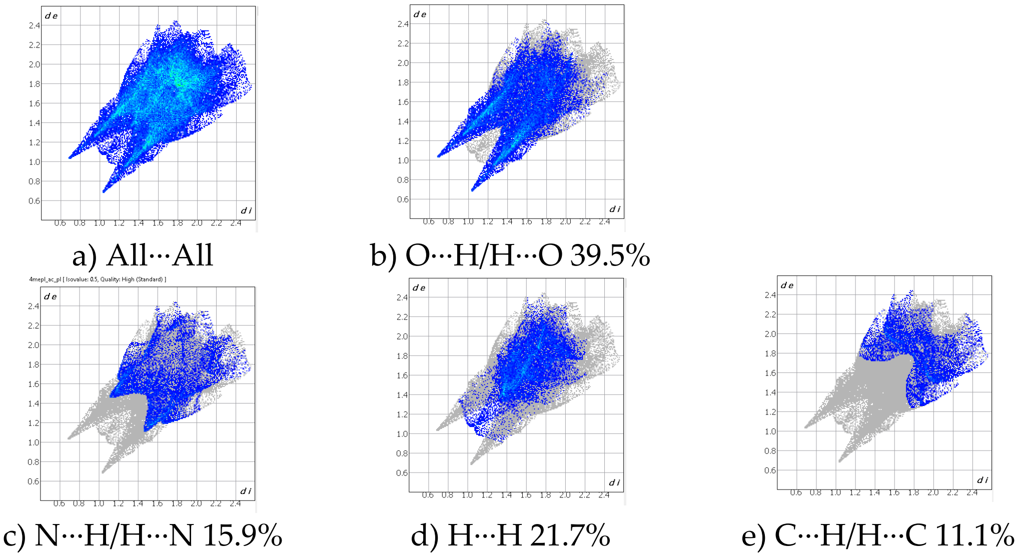

Figure S4. full two-dimensional fingerprint plots for 4-MPTCA showing all interactions.

-247.4 Kj·mol-1

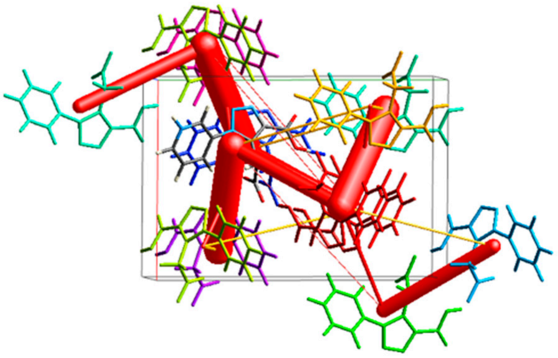

E_ele

-115.0 Kj·mol-1

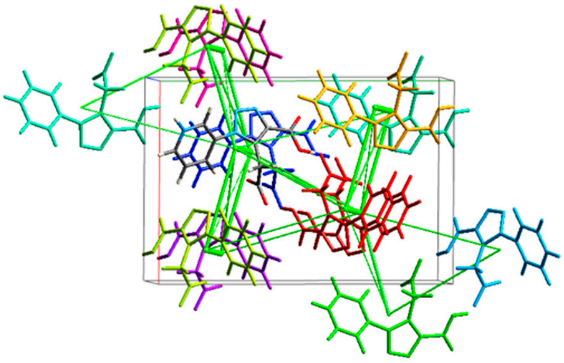

E_dis

-289.8 Kj·mol-1

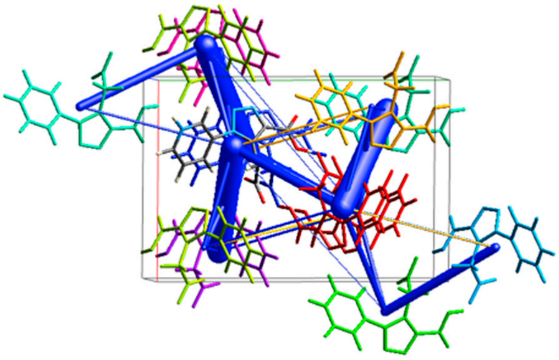

E_tot

Figure S5. Energy framework of 3-PTCA

16.03 Kj·mol-1

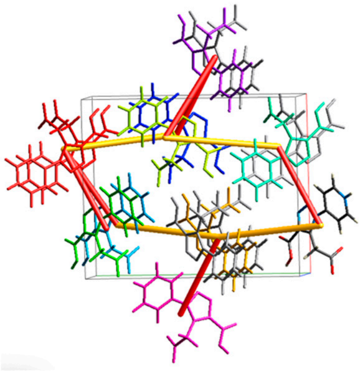

E_ele

-24.42 Kj·mol-1

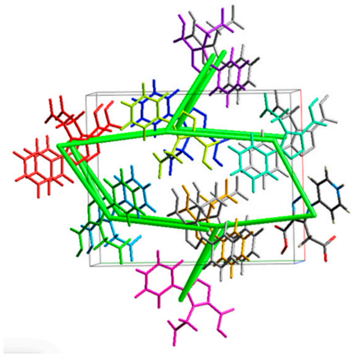

E_dis

-4.58 Kj·mol-1

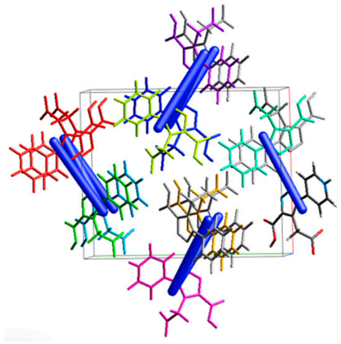

E_tot

Figure S6. Energy framework of 4-PTCA

-299.38 Kj·mol-1

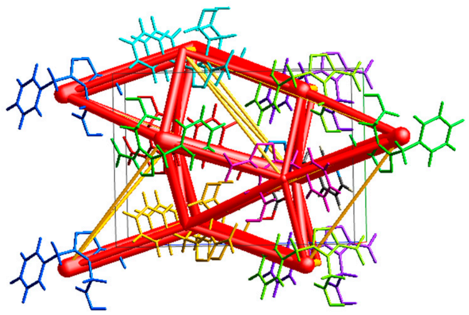

E_ele

-141.80 Kj·mol-1

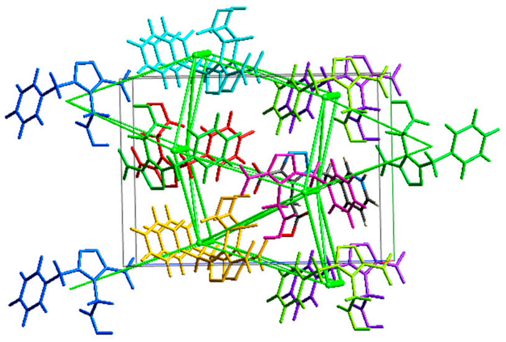

E_dis

-400.93 Kj·mol-1

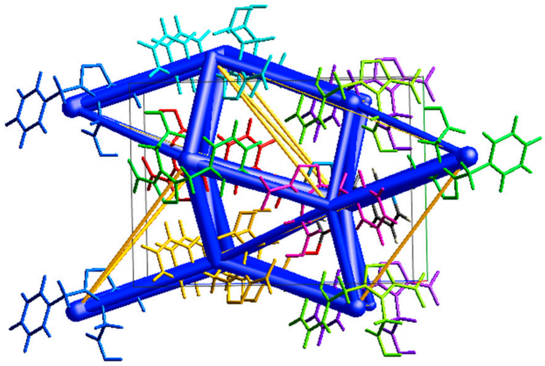

E_tot

Figure S7. Energy framework of 3-MPTCA

-197.78 Kj·mol-1

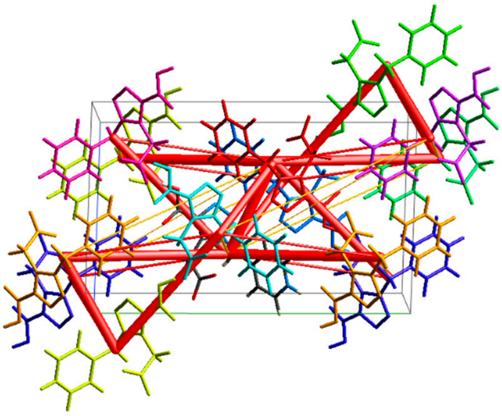

E_ele

-153.60 Kj·mol-1

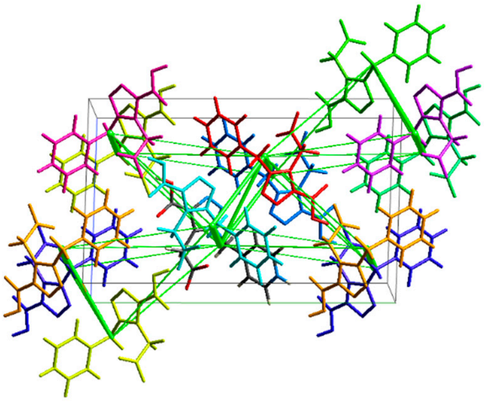

E_dis

-284.02 Kj·mol-1

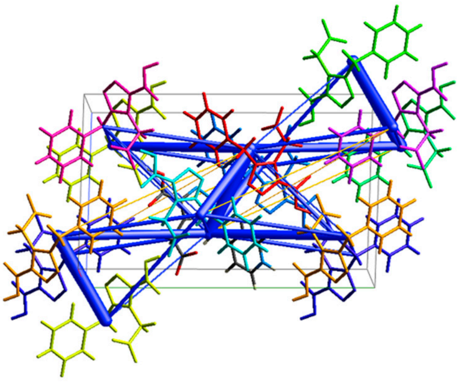

E_tot

Figure S8. Energy framework of 4-MPTCA

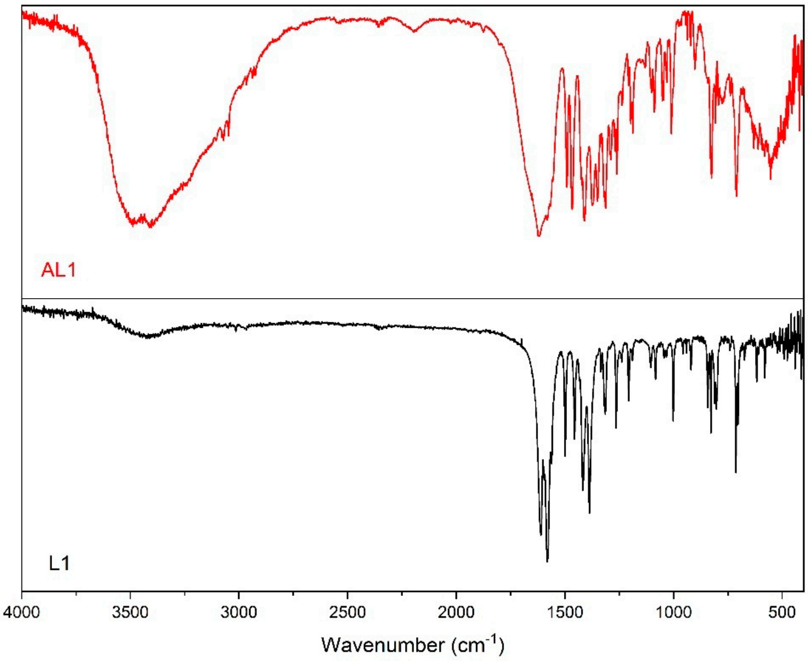

Figure S9. FT-IR Spectra of 3-PTCA (black) and ligand (red)

^{1}H NMR (300 MHz, DMSO-d_6) δ 8.82 (dd, *J* = 4.8, 1.5 Hz, 1H, H2), 8.79 (d, *J* = 2.5 Hz, 1H, H4), 8.07 (ddd, *J* = 8.2, 2.6, 1.5 Hz, 1H, H5), 7.71 (dd, *J* = 8.2, 4.8 Hz, 1H, H6), 4.09 (s, 1H, -CH_2-).

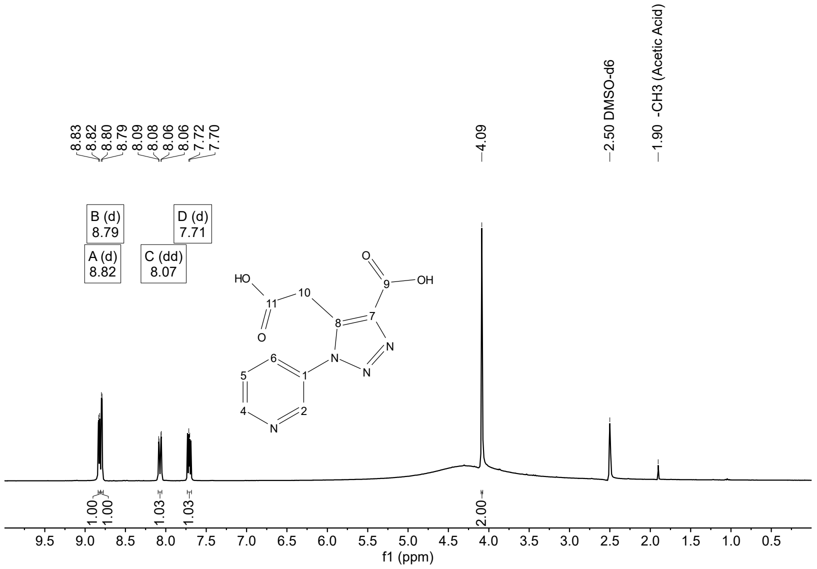

^{13}C NMR (75 MHz, DMSO) δ 169.31 (C11), 162.23(C9), 151.45(C4), 146.03 (C2), 137.92 (C9-*ipso*), 137.29 (C1-*ipso*), 133.51(C6), 132.19(C8-*ipso*), 124.77(C5), 29.69(-CH_3 Acetic acid).

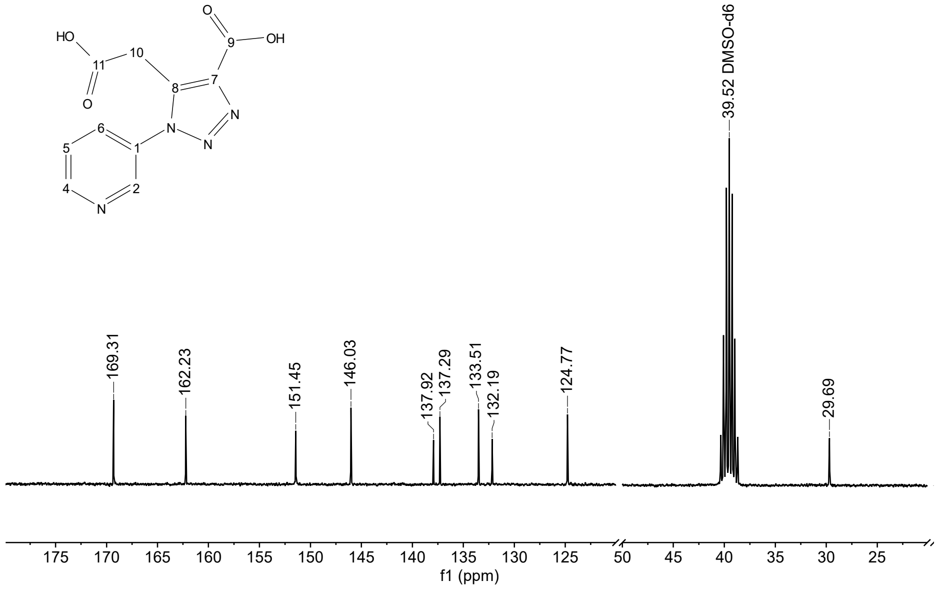

Figure S10. ^{1}H-NMR and ^{13}C-NMR spectra of 3-PTCA

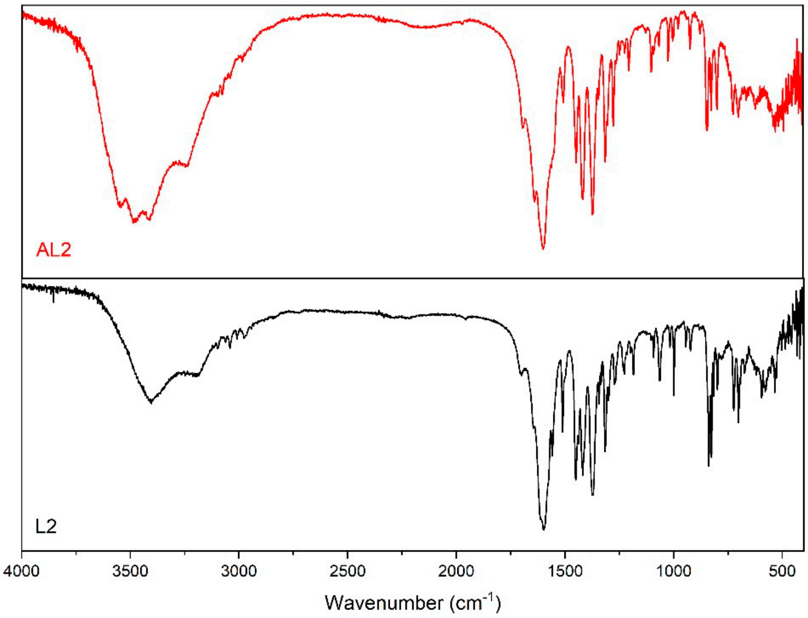

Figure S11. FT-IR Spectra of 4-PTCA (black) and ligand (red)

^{1}H NMR (300 MHz, DMSO) δ 2.51 (d, *J* = 6.1 Hz, 7H), 4.20 (s, 1H), 7.70 (d, *J* = 5.2 Hz, 1H), 8.88 (d, *J* = 5.0 Hz, 1H).

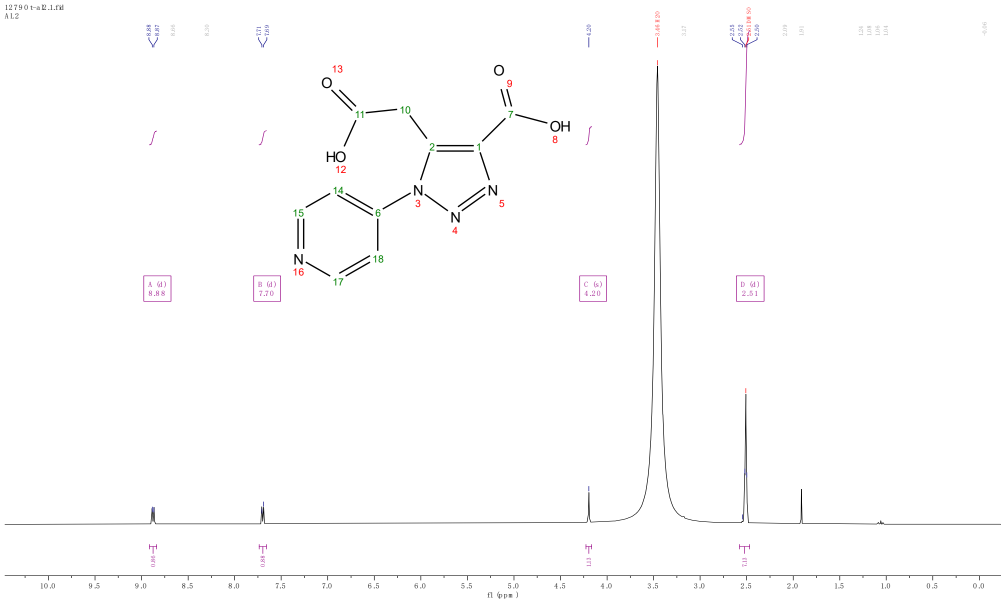

^{13}C NMR (75 MHz, DMSO) δ 30.16 (C10), 119.60 (C14, C18), 137.15 (C1, C2), 142.68 (C6), 152.12 (C15, C17), 162.48 (C7), 169.58 (C11).

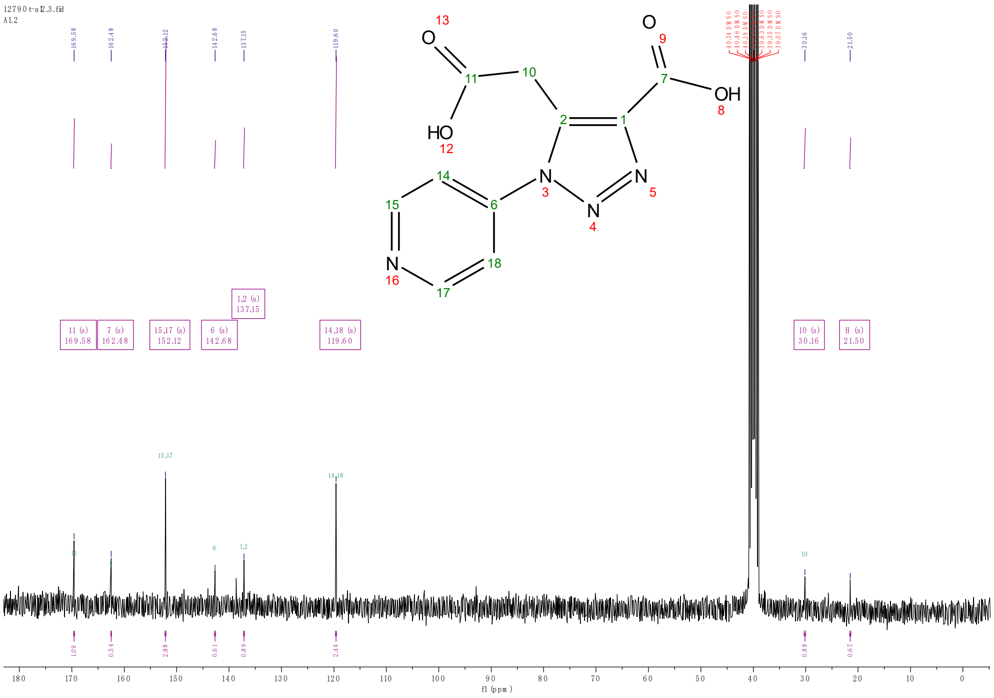

Figure S12. ^{1}H-NMR and ^{13}C-NMR spectra of 4-PTCA

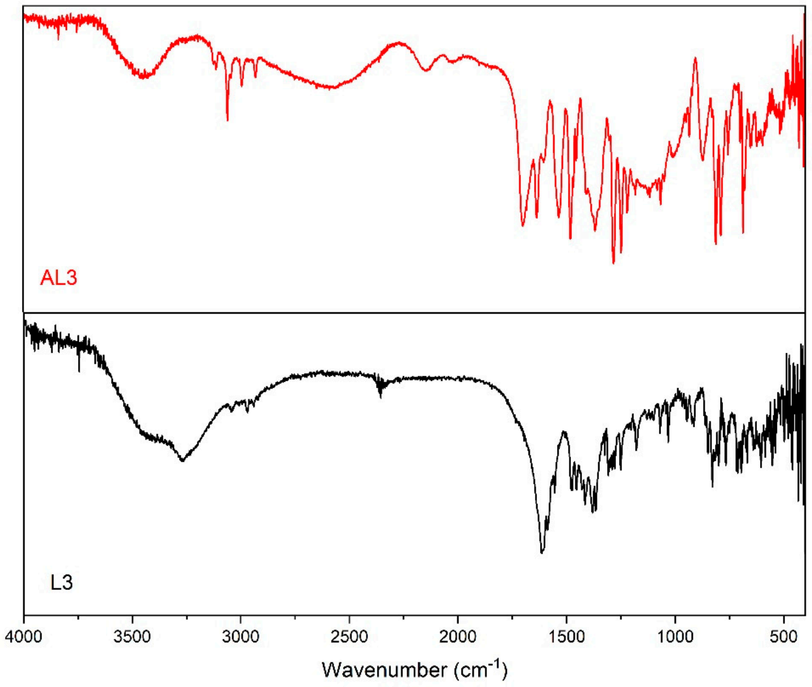

Figure S13. FT-IR Spectra of 3-MPTCA (black) and ligand (red)

^{1}H NMR (300 MHz, DMSO) δ 4.21 (s, 2H, 16), 5.76 (s, 2H, 6), 7.44 (ddd, *J* = 7.9, 4.8, 0.8 Hz, 1H, 11), 7.73 (dt, *J* = 7.9, 2.0 Hz, 1H, 12), 8.54 – 8.65 (m, 2H, 13, 15).

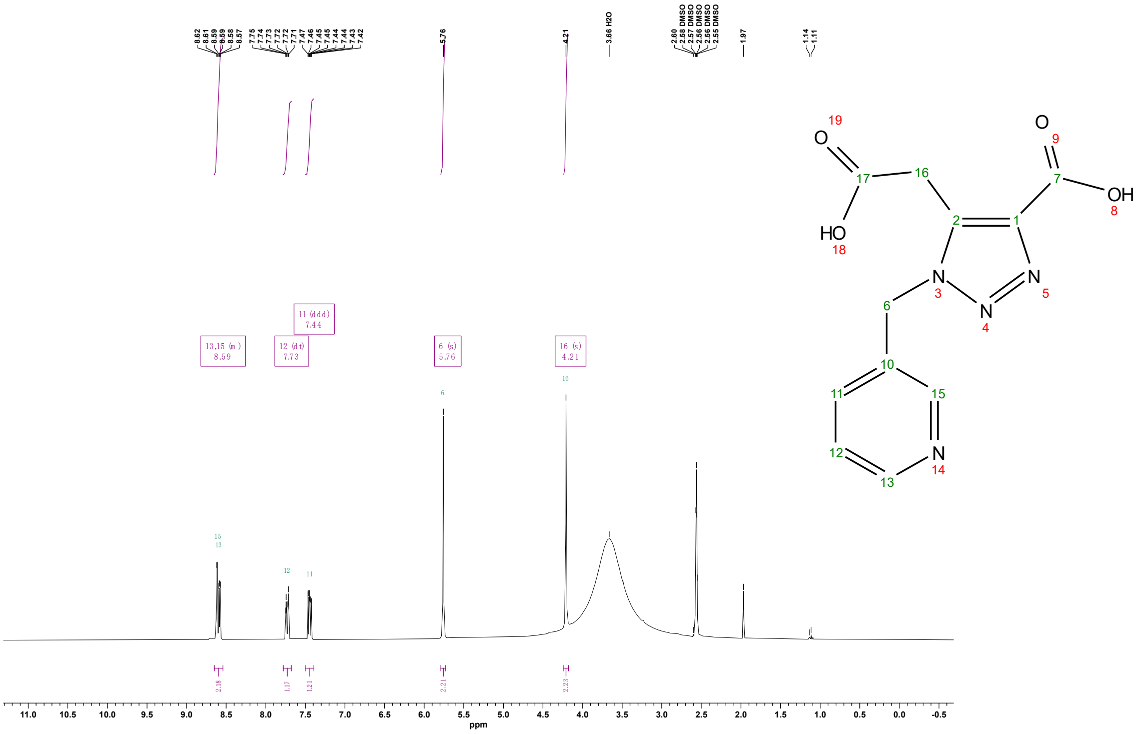

^{13}C NMR (75 MHz, DMSO) δ 169.66 (C17), 162.66 (C7), 149.70 (C15), 149.54 (C13), 137.93 (C11), 136.86 (C1), 136.34 (C2), 131.45 (C10), 124.18 (C12), 48.90 (C6), 29.55 (C16).

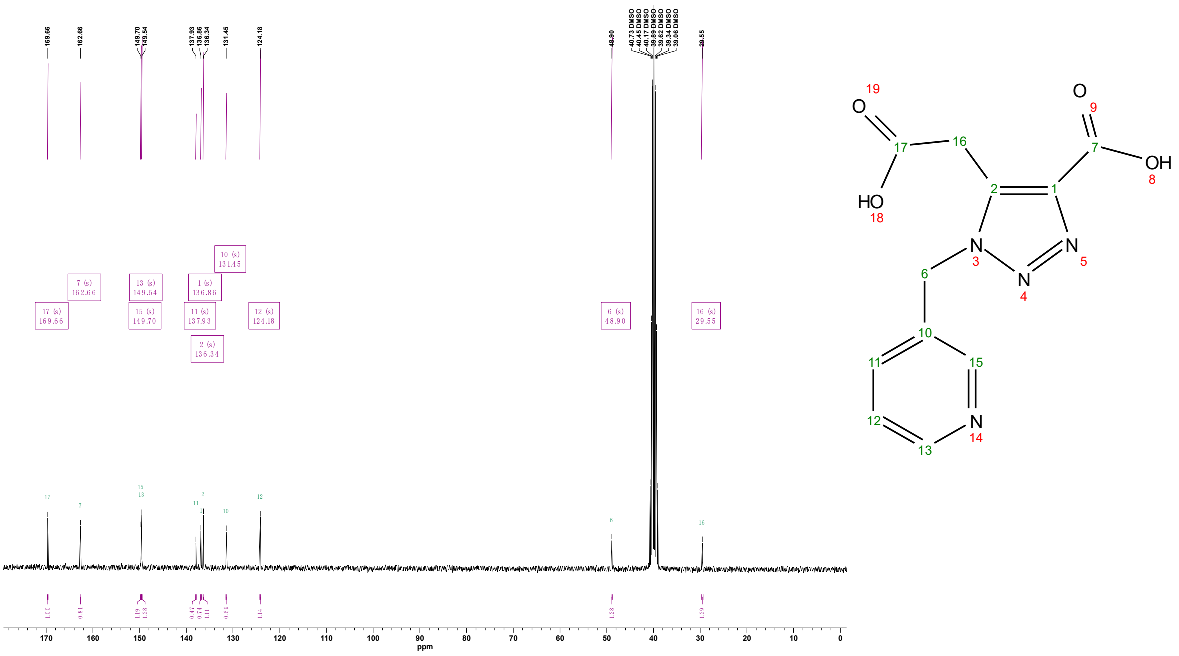

Figure S14. ^{1}H-NMR and ^{13}C-NMR spectra of 3-MPTCA

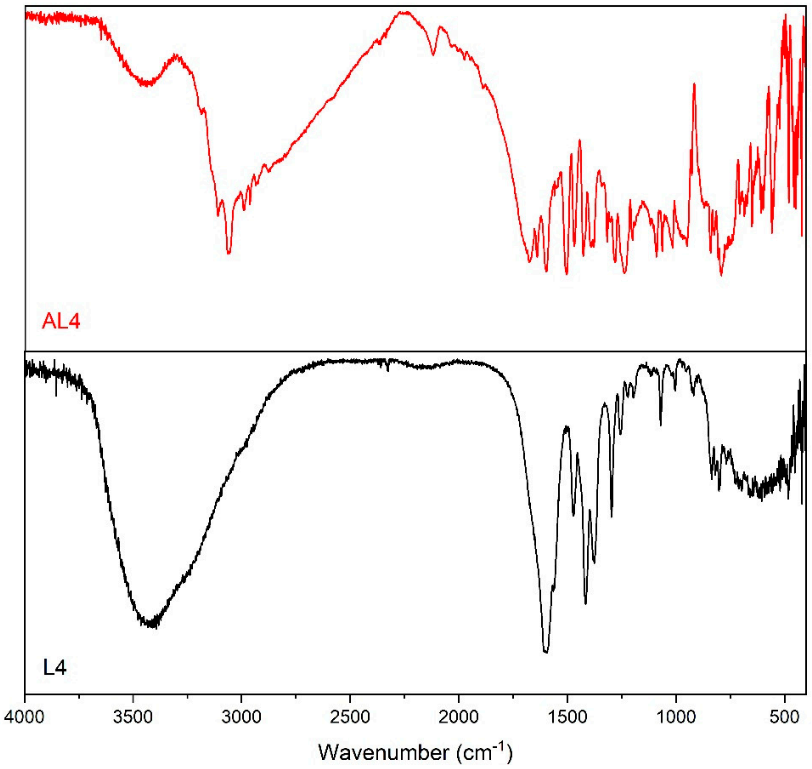

Figure S15. FT-IR Spectra of 4-MPTCA (black) and ligand (red)

^{1}H NMR (300 MHz, DMSO) δ 3.87 (s, 8H, 16), 4.15 (s, 0H), 5.80 (s, 0H, 6), 7.20 – 7.28 (m, 0H, 12, 14), 8.55 – 8.64 (m, 0H, 11, 15).

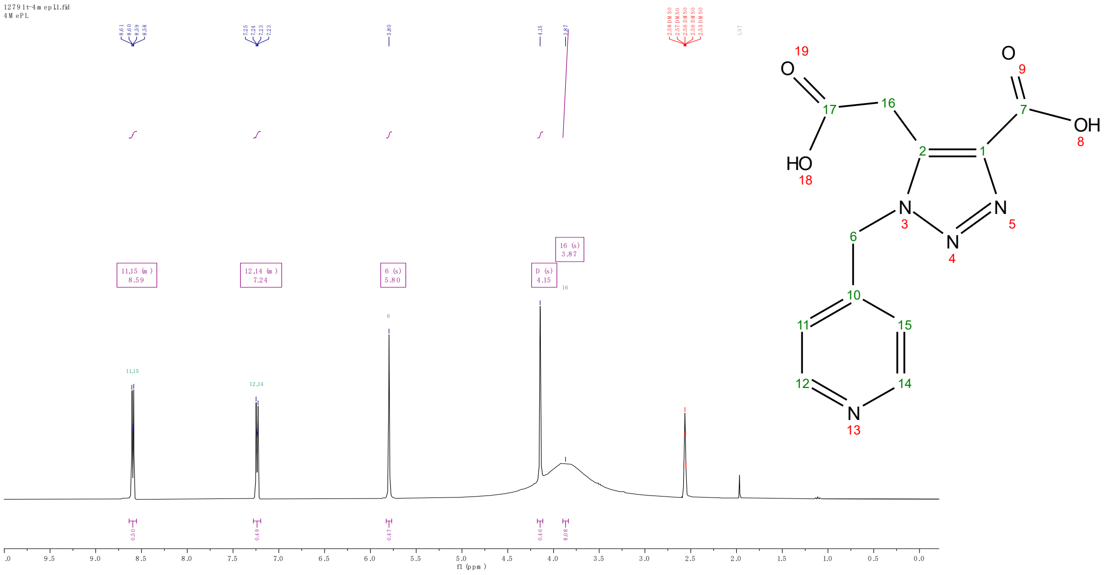

^{13}C NMR (75 MHz, DMSO) δ 29.55 (C16), 50.06 (C6), 122.79 (C11, C15), 137.18 (C2), 138.05 (C1), 144.63 (C10), 150.27 (C12, C14), 162.65 (C7), 169.58 (C17).

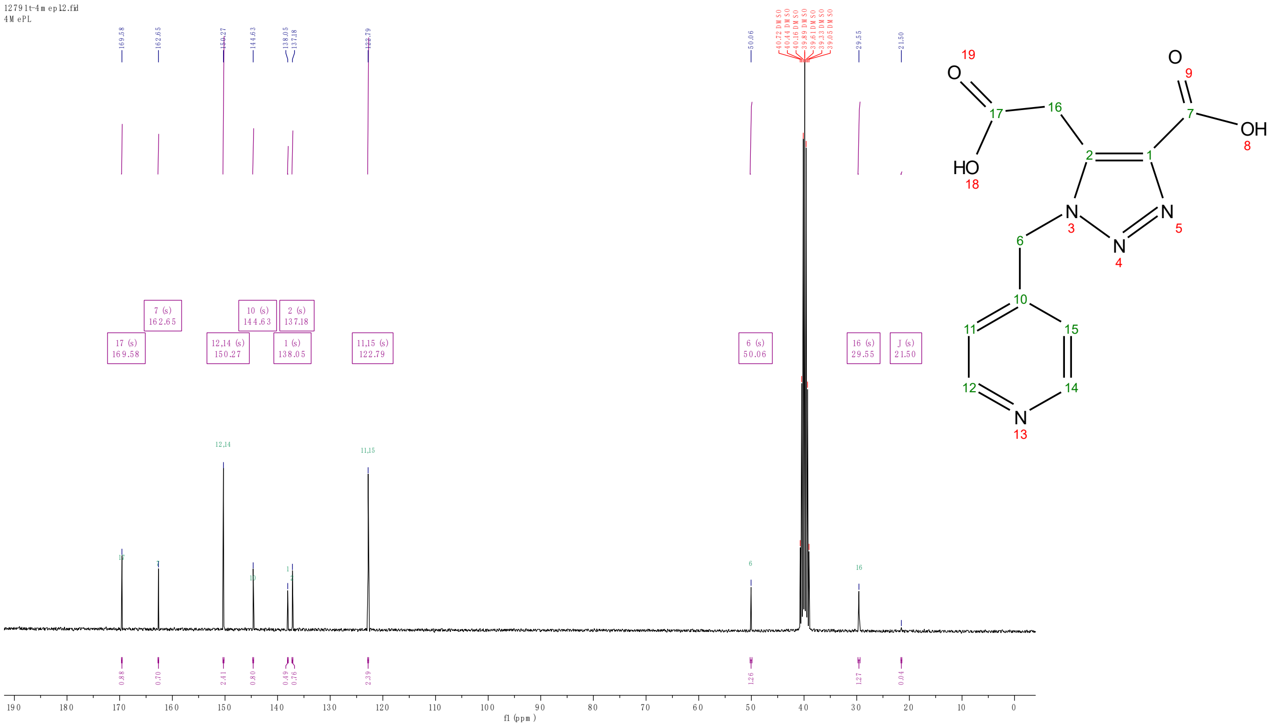

Figure S16. ^{1}H-NMR and ^{13}C-NMR spectra of 4-MPTCA

Table S1. Total energy force diagrams and the details of interaction with symmetry operation (Symop) and color-coded interaction distances between molecular centroids (R) in Å for 3-PTCA

N	Symop	R	E_ele	E_pol	E_dis	E_rep	E_tot
2	-x, -y, z+1/2	7.94	-85.55	-32.5	-13.8	113.2	-56.61
1	-x+1/2, y+1/2, z+1/2	9.42	14.07	-0.95	-1.19	0	13.14
2	x+1/2, -y+1/2, z	6.49	-0.41	-25.5	-25.3	19.37	-29.36
1	-x+1/2, y+1/2, z+1/2	13.56	-5.44	-0.46	-0.21	0	-6.27
2	-x+1/2, y+1/2, z+1/2	9.42	-3.47	-6	-11.2	2.91	-16.06
1	x+1/2, -y+1/2, z	16.39	2.99	-0.04	-0.04	0	3.1
1	x, y, z	5.03	17.7	-26.6	-41.1	27.78	-19.61
1	x+1/2, -y+1/2, z	8.21	-164.4	-47.9	-13.8	125.5	-143.7
1	x+1/2, -y+1/2, z	8.21	-22.94	-7.44	-8.65	4.64	-34.42
TOTAL			-247.4	-147	-115	293.4	-289.8

Table S2. Total energy force diagrams and the details of interaction with symmetry operation (Symop) and color-coded interaction distances between molecular centroids (R) in Å for 4-PTCA

N	Symop	R	E_ele	E_pol	E_dis	E_rep	E_tot
1	-x, -y, z+1/2	21.2	0.48	-0.01	-0.01	0	0.49
1	-x+1/2, y+1/2, z+1/2	8.81	11.75	-7.39	-11.62	5.22	0.06
1	x+1/2, -y+1/2, z	13.5	2.97	-0.09	-0.16	0	2.94
1	x, y, z	17	2.56	-0.04	-0.03	0	2.64
1	-x, -y, z+1/2	7.5	-11.2	-8.18	-12.29	13.61	-20.2
1	x, y, z	17.6	0.44	-0.05	-0.03	0	0.4
1	x+1/2, -y+1/2, z	14.2	3.28	-0.07	-0.1	0	3.33
1	-x+1/2, y+1/2, z+1/2	16.1	4.17	-0.15	-0.07	0	4.23
1	x+1/2, -y+1/2, z	14.2	1.56	-0.07	-0.11	0	1.5
TOTAL			16.03	-16.05	-24.42	18.83	-4.58

Table S3. Total energy force diagrams and the details of interaction with symmetry operation (Symop) and color-coded interaction distances between molecular centroids (R) in Å for 3-MPTCA

N	Symop	R	E_ele	E_pol	E_dis	E_rep	E_tot
1	-x+1/2, -y, z+1/2	9.39	-54.24	-17.45	-16.1	6.21	-80.45
2	x+1/2, -y+1/2, -z	8.21	-71.76	-32.27	-15.98	95.96	-54.4
2	-x, y+1/2, -z+1/2	7.08	-73.7	-22.96	-21.95	19.35	-102.07
2	-x+1/2, -y, z+1/2	10.35	-132.55	-34.71	-8.77	67.5	-131.76
2	x+1/2, -y+1/2, -z	11.60	24.82	-1.89	-0.65	0	24.28
2	-x, y+1/2, -z+1/2	17.24	7.06	-0.17	-0.04	0	7.3
2	-x, y+1/2, -z+1/2	7.32	-51.96	-13.43	-24.12	24.6	-70.68
1	x, y, z	4.75	52.95	-39.26	-54.19	43.89	6.85
TOTAL			-299.38	-162.14	-141.8	257.51	-400.93

Table S4. Total energy force diagrams and the details of interaction with symmetry operation (Symop) and color-coded interaction distances between molecular centroids (R) in Å for 4-MPTCA

N	Symop	R	E_ele	E_pol	E_dis	E_rep	E_tot
1	-x, -y, -z	6.35	-58.74	-18.76	-27.77	27.3	-83.3
2	-x, y+1/2, -z+1/2	10.05	-57.98	-17.75	-10.27	38.22	-59.77
2	x, -y+1/2, z+1/2	8.40	3.54	-8.92	-11.98	3.79	-10.95
1	-x, y+1/2, -z+1/2	14.36	-0.37	-0.05	-0.15	0	-0.56
1	x, -y+1/2, z+1/2	14.44	9.13	-0.62	-0.29	0	8.95
1	x, y, z	4.95	16.29	-29.06	-48.86	41.63	-21.11
1	-x, -y, -z	6.30	-48.05	-14.47	-32.34	31.07	-70.48
2	-x, y+1/2, -z+1/2	9.83	-14.97	-6.36	-8.45	1.21	-27.15
1	x, -y+1/2, z+1/2	15.14	5.88	-0.37	-0.18	0	5.79
1	x, -y+1/2, z+1/2	9.56	-52.51	-29.66	-13.31	102.97	-25.44
TOTAL			-197.78	-126.02	-153.6	246.19	-284.02
